# Role of Plant Growth Promoting Rhizobacteria in Agricultural Sustainability—A Review

**DOI:** 10.3390/molecules21050573

**Published:** 2016-04-29

**Authors:** Pravin Vejan, Rosazlin Abdullah, Tumirah Khadiran, Salmah Ismail, Amru Nasrulhaq Boyce

**Affiliations:** 1 Institute of Biological Sciences, Faculty of Science, University of Malaya, 50603 Kuala Lumpur, Malaysia; pravin92@siswa.um.edu.my (P.V.); salmah_r@um.edu.my (S.I.); amru@um.edu.my (A.N.B.); 2 Centre for Research in Biotechnology for Agriculture (CEBAR), Institute of Biological Sciences, Faculty of Science, University of Malaya, 50603 Kuala Lumpur, Malaysia; 3 Forest Product Divisions, Forest Research Institute Malaysia, 52109 Kepong, Selangor, Malaysia; tumirah@frim.gov.my

**Keywords:** biofertilizer, plant growth promoting rhizobacteria (PGPR), plant-microbes, plant growth, nano-encapsulation technology

## Abstract

Plant growth promoting rhizobacteria (PGPR) shows an important role in the sustainable agriculture industry. The increasing demand for crop production with a significant reduction of synthetic chemical fertilizers and pesticides use is a big challenge nowadays. The use of PGPR has been proven to be an environmentally sound way of increasing crop yields by facilitating plant growth through either a direct or indirect mechanism. The mechanisms of PGPR include regulating hormonal and nutritional balance, inducing resistance against plant pathogens, and solubilizing nutrients for easy uptake by plants. In addition, PGPR show synergistic and antagonistic interactions with microorganisms within the rhizosphere and beyond in bulk soil, which indirectly boosts plant growth rate. There are many bacteria species that act as PGPR, described in the literature as successful for improving plant growth. However, there is a gap between the mode of action (mechanism) of the PGPR for plant growth and the role of the PGPR as biofertilizer—thus the importance of nano-encapsulation technology in improving the efficacy of PGPR. Hence, this review bridges the gap mentioned and summarizes the mechanism of PGPR as a biofertilizer for agricultural sustainability.

## 1. Introduction

Agriculture is one of the human activities that contributes most to the increasing amount of chemical pollutants via excessive use of synthetic chemical fertilizers and pesticides, which cause further environmental damage with potential risks to human health. Nitrous oxide (N_2_O) is an example of chemical pollutant produced by excessive use of nitrogen fertilizer and is a major source of greenhouse gases causing global warming. Moreover, 74% of total U.S. N_2_O emissions in 2013 were accounted for by agricultural soil management, the largest single source [1]. Apart from that, nitrogen fertilizers reduce biological nitrogen fixation in the soil. Farmers apply a high concentration of nitrogen fertilizers in the form of ammonium nitrate to fertilize their soil to grow crops. Due to the influx of ammonium, plants no longer need the symbiotic microbes to provide ammonium and this leads to the degree of symbiosis being diminished. Furthermore, nitrifying bacteria also take advantage of this excess ammonium and utilize it to produce nitrate. This high amount of nitrate is then utilized by denitrifying bacteria to produce N_2_O and excess nitrate leaches into the groundwater [2]. As a result, increased microbial processes of nitrification and denitrification increase the natural production of N_2_O. Denitrification is the step whereby nitrogen oxides are reduced by microorganisms to gaseous products and released back into the atmosphere and nitrification is a two-step process of ammonium (NH_4_) being converted to nitrate (NO_3_) by soil bacteria [3].

Towards a sustainable agricultural vision, crops produced need to be equipped with disease resistance, salt tolerance, drought tolerance, heavy metal stress tolerance, and better nutritional value. To fulfil the above desired crop properties, one possibility is to use soil microorganisms (bacteria, fungi, algae, *etc.*) that increase the nutrient uptake capacity and water use efficiency [4]. Among these potential soil microorganisms, bacteria known as plant growth promoting rhizobacteria (PGPR) are the most promising. In this sense, PGPR may be used to enhance plant health and promote plant growth rate without environmental contamination [5].

For decades, varieties of PGPR have been studied and some of them have been commercialized, including the species *Pseudomonas*, *Bacillus*, *Enterobacter*, *Klebsiella*, *Azobacter*, *Variovorax Azosprillum*, and *Serratia* [6]. However, the utilization of PGPR in the agriculture industry represents only a small fraction of agricultural practice worldwide [7]. This is due to the inconsistent properties of the inoculated PGPR, which could influence crop production. The successful utilization of PGPR is dependent on its survival in soil, the compatibility with the crop on which it is inoculated, the interaction ability with indigenous microflora in soil, and environmental factors [8]. Another challenge is that the modes of action of PGPR are diverse and not all rhizobacteria possess the same mechanisms [9,10]. These disadvantages limit the application of PGPR. Therefore, the competition between synthetic chemical fertilizers and PGPR as a biofertilizer is deemed redundant in the face of the global agricultural productivity needed to feed the booming world’s population, which is predicted to escalate to 8 billion people by 2025 and 9 billion by 2050.

According to Nakkeeran *et al.* [11], an ideal PGPR should possess high rhizosphere competence, enhance plant growth capabilities, have a broad spectrum of action, be safe for the environment, be compatible with other rhizobacteria, and be tolerant to heat, UV radiation, and oxidizing agent. Considering the factors discussed above, the need for a better PGPR biofertilizer to complement skyrocketing agricultural food production as one of the crucial drivers of the economy has been highlighted. The inclusion of nano-encapsulation technology has been vital to the revolution of today’s PGPR biofertilizers’ formulation.

This review will therefore attempt to shed more light on the modes of action of PGPR, the role of PGPR as biofertilizer, and the advantages of nano-encapsulation technology towards PGPR as a biofertilizer. The information generated from this review could be very beneficial to those who are concerned about environmental protection and agricultural sustainability.

## 2. Plant Growth Promoting Rhizobacteria

Plant growth promoting rhizobacteria (PGPR) is a group of bacteria that can be found in the rhizosphere [12]. The term “plant growth promoting bacteria” refers to bacteria that colonize the roots of plants (rhizosphere) that enhance plant growth. Rhizosphere is the soil environment where the plant root is available and is a zone of maximum microbial activity resulting in a confined nutrient pool in which essential macro- and micronutrients are extracted. The microbial population present in the rhizosphere is relatively different from that of its surroundings due to the presence of root exudates that function as a source of nutrients for microbial growth [13]. Weller and Thomashow [14] prove that the narrow rhizosphere zone is rich in nutrients for microbes compared to the bulk soil; this is shown by the quantity of bacteria that are present surrounding the roots of the plants, generally 10 to 100 times higher than in the bulk soil.

The microbial colonizing rhizosphere includes bacteria, fungi, acticomycetes, protozoa, and algae. However, bacteria are the most abundant microbial present in the rhizosphere [15]. The enhancement of plant growth by the application of these microbial populations is well known and proven [16,17]. The term “plant growth promoting rhizobacteria (PGPR)” for these beneficial microbes was introduced by Kloepper and Schroth [18], paving the way for greater discoveries on PGPR. PGPR are not only associated with the root to exert beneficial effects on plant development but also have positive effects on controlling phytopathogenic microorganisms [19,20]. Therefore, PGPR serve as one of the active ingredients in biofertilizer formulation.

Based on the interactions with plants, PGPR can be separated into symbiotic bacteria, whereby they live inside plants and exchange metabolites with them directly, and free-living rhizobacteria, which live outside plant cells [21]. The working mechanisms of PGPR can also be separated into direct and indirect ones. The direct mechanisms are biofertilization, stimulation of root growth, rhizoremediation, and plant stress control. On the other hand, the mechanism of biological control by which rhizobacteria are involved as plant growth promotion indirectly is by reducing the impact of diseases, which include antibiosis, induction of systemic resistance, and competition for nutrients and niches [22].

Symbiotic bacteria mostly reside in the intercellular spaces of the host plant, but there are certain bacteria that are able to form mutualistic interactions with their hosts and penetrate plant cells. In addition to that, a few are capable of integrating their physiology with the plant, causing the formation of specialized structures. Rhizobia, the famous mutualistic symbiotic bacteria, could establish symbiotic associations with leguminous crop plants, fixing atmospheric nitrogen for the plant in specific root structures known as nodules. Table 1 summarizes some of the bacteria that have been previously reported as being used as PGPR.

## 3. Role of Plant Growth Promoting Rhizobacteria for Plant Growth Enhancement

PGPR plays an important role in enhancing plant growth through a wide variety of mechanisms. The mode of action of PGPR that promotes plant growth includes (i) abiotic stress tolerance in plants; (ii) nutrient fixation for easy uptake by plant; (iii) plant growth regulators; (iv) the production of siderophores; (v) the production of volatile organic compounds; and (vi) the production of protection enzyme such as chitinase, glucanase, and ACC-deaminase for the prevention of plant diseases [10,23]. However, the mode of action of different PGPR varies depending on the type of host plants [9].

Plant growth is influenced by a variety of stresses due to the soil environment, which is a major constraint for sustainable agricultural production. These stresses can be classified into two groups, biotic and abiotic. Biotic refers to the stresses due to plant pathogens and pests such as viruses, fungi, bacteria, nematodes, insects, *etc.*, while abiotic is stresses due to the content of heavy metal in soils, drought, nutrient deficiency, salinity, temperature, and so on.

### 3.1. Abiotic Stress Tolerance in Plants

Abiotic stresses are considered to be the main sources of agricultural yield reduction. However, the intensity of abiotic stress varies depending on the type of soils (deficiency of hormonal and nutritional imbalances) and plant factors (physiological disorders such as being susceptible to diseases, abscission, *etc.*) [24]. The PGPR mechanisms in plant towards abiotic stress were previously studied extensively. Pishchik *et al.* [25] reported that PGPR could be attenuated by the toxic effect of cadmium pollution on barley plants due to the ability of the bacteria to cadmium ions from the soil by binding mechanisms, thereby decreasing the availability of cadmium in the soil.

Moreover, Nautiyal *et al.* [26] demonstrated that the *Bacillus lentimorbus* strain increased the antioxidant capacity of the edible parts of spinach, carrots, and lettuce, as well as increasing growth. The results produced are important, especially to improve the nutrient content of these crops.

Another major effect of PGPR on plants under abiotic stress conditions is the improvement of leaf water status, especially under salinity and drought stress [55,67]. Sarma and Saikia [68] reported that *Pseudomonas aeruginosa* strain has improved the growth of *Vigna radiata* (mung beans) plants under drought conditions. The ability of plants in utilizing water for growth depends on their stomatal apertures. The stomatal on the plant leaf functions to balance the water content in leaf and water uptake by the roots. Ahmad *et al.* [55] and Naveed *et al.* [67] reported that the stomatal conductance (water vapor exiting through the stomata leaf) of plant leaf was higher in PGPR inoculated plants than non-PGPR inoculated ones under drought conditions. The finding from both studies proves that PGPR-inoculated plants tend to improve the water-use efficiency of plants. This finding could be beneficial to the environment in terms of reducing excessive usage of water.

Marulanda *et al.* [69] reported that *Bacillus megatertum* strain inoculated into maize roots increased the ability of the root to absorb water under the salinity conditions. Gond *et al.* [70] also found similar behavior when *Pantoea agglomerans* was inoculated into the maize roots. They found that the ability of the maize root to absorb water in saline conditions has improved. Here, bacteria that can grow under hypersaline conditions will be better able to colonize the root rhizospheres and external spaces of roots that are themselves exposed to high salinity conditions. Thus, the strategy was to first screen the bacterial isolates for their ability to grow under hypersaline conditions.

Gonzalez *et al.* [71] used *Azospirillum brasilense* to improve the salt tolerance of the jojoba plant during *in vitro* rooting. Based on the findings obtained, *A. brasilense* can reduce the undesirable effects of saline conditions on the jojoba rooting. The bacteria attenuated salinity’s effect on the rooting ability of the jojoba plant. This indicates that *A. brasilense* has higher plant tolerance to salt stress.

Gabriela *et al.* [72] also used *Azospirillum* to study lettuce growth under salt stress. They found that inoculation with *Azospirillum* sp. not only improves lettuce quality but also extends the storage life of a lettuce grown under salt stress, which further improves the yield.

### 3.2. Nutrient Availability for Plant Uptake

PGPR has the ability to increase the availability of nutrient concentration in the rhizosphere [10] by fixing nutrients, thus preventing them from leaching out. As an example, nitrogen, which is needed for the synthesis of amino acids and proteins, is the most limiting nutrient for plants. The mechanisms by which atmospheric nitrogen is added into organic forms that can be assimilated by plants are exclusive to prokaryotes [73,74]. A rare example of a free-living nitrogen-fixing organism is *Azospirillum*, often associated with cereals in temperate zones and also reported to be able to improve rice crop yields [31].

Some PGPR have the ability to solubilize phosphate [75], resulting in an increased availability of phosphate ions in the soil, which can be easily taken up by the plants. *Kocuria turfanensis* strain 2M4 isolated from rhizospheric soil was discovered to be a phosphate solubilizer, an IAA producer, and a siderophore producer [76].

Lavakush *et al.* [77] studied the effect of PGPR on nutrient uptake by rice. They used PGPR strains such as *Pseudomonas fluorescens*, *Pseudomonas putida*, and *Pseudomonas fluorescens*.

### 3.3. Plant Growth Regulators

These plant growth regulators, also termed plant exogenous hormones, are synthetic substances that are similar to natural plant hormones. They are used to regulate the growth of plants and are important measures for boosting agricultural production. One of the terms for the prominent modes of action for growth promotion by PGPR is phytostimulator, or plant growth regulator. This is defined as microorganisms that have the ability to produce or alter the concentration of growth regulators such as IAA, GA, cytokinins, and ethylene. The mechanism that is being projected is the production of phytohormones (plant hormones) such as auxins, cytokinins, and GA [78,79]. Phytohormones are organic substances found in extremely low amounts that exert influence on the biochemical, physiological, and morphological processes in plants; their synthesis is smoothly regulated. Phytohormones that are not naturally synthesized by the plants but are synthesized exogenously by natural and synthetic means are known as plant growth regulators. The following examples are phytohormones that are synthesized directly and indirectly by PGPR, which act as plant growth regulators.

Auxin is one of the crucial molecules, regulating most plant processes directly or indirectly [80] as was further proven when Ahmed and Hasnain [35] reported that auxin-producing *Bacillus* spp. inflicts a positive effect on *Solanun tuberosum*’s growth. The most active and famous auxins in plants is indole-3-acetic acid (IAA) [81]. According to Spaepen and Vacheron [82,83], a wide range of processes in plant development and plant growth are controlled by exogenous IAA in which a low amount of IAA can stimulate primary root elongation, whereas high IAA levels decrease primary root length, increase root hair formation, and stimulate the formation of lateral roots. Thus, plants have greater access to soil nutrients as bacterial IAA increases both the root surface area and length. The processes of seed germination and emergence, floral induction, flower and fruit development, and steam and leaf growth include the involvement of gibberellin (GA), which is one of the phytohormones [84]. However, the most dominant physiological effect of GA is shoot elongation [85]. Khan [65] showed that tomato plants inoculated with the gibberellin-producing *Sphingomonas* sp. LK11 strain have a significant increase in various growth characteristics. Cytokinins stimulate a plant’s cell division, vascular cambium sensitivity, and vascular differentiation and induce the proliferation of root hairs, but inhibit lateral root formation and primary root elongation [28,86]. Liu [87] reported that the oriental Thuja seedlings inoculated with cytokinin-producing *Bacillus subtilis* strains were more resistant to stress due to draught.

**Figure 1:**
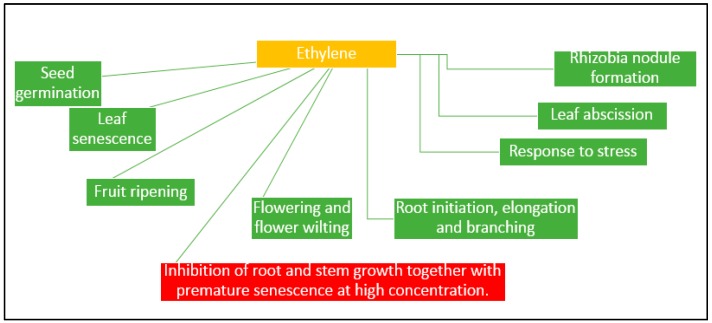
The phytohormone ethylene affects a large number of different processes in the growth and development of a plant.

Ethylene is another plant hormone known to regulate many processes such as the ripening of fruits, the abscission of leaves, or the ripening of fruits (Figure 1) [88]. Moreover, at high concentrations, ethylene induces the defoliation and cellular processes that lead to the inhibition of root and stem growth together with premature senescence, all of which lead to poorer crop performance [89]. The plants synthesized 1-aminocyclopropane-1-carboxylate (ACC), which is the precursor for ethylene, in response to exposure to various types of environmental stress, such as cold, drought, flooding, infections with pathogens, and the presence of heavy metals [90]. High levels of ethylene, produced under stress conditions, can halt certain processes such as root elongation or nitrogen fixation in legumes [91], and cause premature senescence [55].

Here, PGPR with the action to degrade ACC in the rhizosphere could shorten the deteriorating cycle and reconstruct a healthy root system that would withstand environmental stress. Furthermore, Glick [92] has illustrated how plant growth-promoting bacteria that produce ACC deaminase and synthesize IAA may facilitate plant growth. Enzyme ACC deaminase involved in the primary mechanism rhizobacteria is utilized to degrade ethylene [92]. Ahmad [55] proved that *Rhizobium* and *Pseudomonas* ACC-deaminase-producing strains can improve the growth, physiology, and quality of mung beans under salt-affected environments.

### 3.4. Production of Hormones

Plant hormones are chemical messengers that influence the plant’s ability to react to its environment. These are naturally organic compounds that are effective at very low concentration and are mostly synthesized in certain parts of the plant and transported to another location. Plant hormones, also referred to as phytohormones, influence physiological processes at low concentrations. The influenced processes include growth, differentiation, and development; other processes, such as stomatal movement, could also be affected [93]. It is also important to note that every plant response is often the result of two or more hormones acting together. Thus, since hormones stimulate or inhibit plant growth, they are also referred to as plant growth regulators that are produced from PGPR [94]. A few notable plant hormones such as auxins, ethylene, gibberellins, (+)-abscisic acid (ABA), and cytokinins may well regulate plant growth and development [95,96].

### 3.5. Production of Siderophores

**Figure 2:**
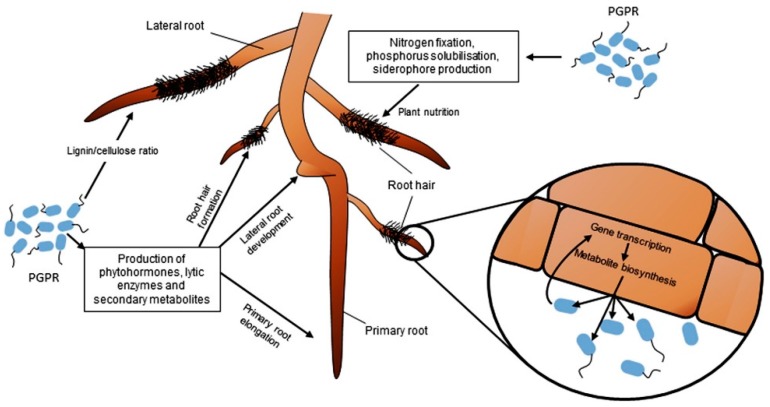
The possible mode of action used by plant growth promoting rhizobacteria (PGPR) towards growth promotion in plants. The flow and location of nitrogen fixation, phosphorus solubilization, and siderophore production are shown [83].

Iron is among the bulk minerals present on the surface of the earth, yet it is unavailable in the soil for plants. Iron is commonly present in nature in the form of Fe^3+^, which is highly insoluble; to solve this problem, PGPR secrete siderophores. Siderophores are low molecular weight iron binding protein compounds involved in the process of chelating ferric iron (Fe (iii)) from the environment. When Fe is limited, microbial siderophores provide plants with Fe, enhancing their growth. Flores-Felix [53] showed that a siderophore-producing *Phyllobacterium* strain promotes the growth and quality of strawberries. Here, plants sequester iron by utilizing siderophores secreted by the mentioned PGPR. The predicted flow of this mode of action is shown in Figure 2.

### 3.6. Production of Volatile Organic Compound

Volatile organic compounds (VOCs) produced by plant growth promoting rhizobacteria (PGPR) are heavily involved in improving plant growth and induce systemic resistance (ISR) towards pathogens [1e3]. Several bacterial species, from diverse genera including *Bacillus, Pseudomonas, Serratia, Arthrobacter*, and *Stenotrophomonas*, produce VOCs that influence plant growth. Acetoin and 2,3-butanediol synthesized by *Bacillus* are the best known of these compounds and are responsible for significant improvements in plant growth [97]. Some other PGPR strains emit VOCs that can directly and/or indirectly mediate increases in plant biomass, disease resistance, and abiotic stress tolerance. VOC emission is indeed a common property of a wide variety of soil microorganisms, although the identity and quantity of volatile compounds emitted vary among species [98,99].

### 3.7. Production of Enzymes

In terms of PGPR producing protection enzymes, the mode of action could be labeled that of biopesticides: PGPR promote plant growth through the control of phytopathogenic agents, primarily for the production of metabolites contributing to the antibiosis and antifungal properties used as defense systems. The mechanism would involve the production of hydrolytic enzymes, of which two examples are chitinase and glucanase. Major fungal cell wall components are made up of chitin and beta-glucan, thus chitinases and beta-glucanases producing bacteria would inhibit fungal growth. The *Sinorhizobium fredii* KCC5 and *Pseudomonas fluorescens* LPK2 produce chitinase and beta-glucanases and dictate the fusarium wilt produced by *Fusariumudum* [59]. Apart from exhibiting the production of chitinase and beta-glucanases, *Pseudomonas* spp. inhibits *Rhizoctonia solani* and Phytophthoracapsici, two of the most destructive crop pathogens in the world [54].

## 4. Beneficial and Harmful Aspects of Plant Growth Promoting Rhizobacteria

It is undisputed that rhizobacteria play a crucial role in maintaining soil fertility and upgrading plant growth and development. This growth betterment takes place with the help of several mechanisms as mentioned in previous chapters, although the reverse is true in some other studies [16]. For example, the production of cyanide is known to be a characteristic of certain *Pseudomonas* species. Here, cyanide production by the bacteria is considered as a growth promotion as well as a growth inhibition characteristic. Moreover, cyanide acts as a biocontrol agent against certain plant pathogens [8]; on the other hand, it can also cause adverse effects on plant growth [100]. Vacheron *et al.* [83] stated that auxin production by PGPR can also cause positive as well as negative effects on plant growth. It is important to note that the effectiveness of auxin relies upon its concentration. For instance, at low concentrations, it enhances plant growth, whereas at a high level it inhibits root growth [101].

Furthermore, rhizobitoxine produced by *Bradyrhizobium elkanii* also has a dual effect. Since it is an inhibitor of ethylene synthesis, it can alleviate the negative effect of stress-induced ethylene production on nodulation [102]. On the other hand, rhizobitoxine is also considered a plant toxin because it induces foliar chlorosis in soybeans [103].

So far, the above discussion has proven that although PGPR are very effective at promoting plant growth and development, a select few bacterial species may inhibit growth. However, this negative impact may only occur under certain specific conditions and also by some particular traits. Thus, the selection of a particular strain is of the utmost importance in obtaining maximum benefits in terms of improved plant growth and development.

## 5. Role of Plant Growth Promoting Rhizobacteria as a Biofertilizer

Biofertilizer is becoming a crucial aspect of organic farming and a major player for the economy and for general agricultural production on a global scale. Biofertilizers can be defined as products that contain living microorganisms; when applied to seeds, plant surfaces, or soil, they colonize the rhizosphere or interior of the plant, and promote plant growth by increasing the supply or availability of primary nutrients to the host plant [104]. According to Mishra [105], biofertilizer is a mixture of live or latent cells encouraging nitrogen fixing, phosphate solubilizing, or cellulolytic microorganisms used for applications to soil, seed, roots, or composting areas with the purpose of increasing the quantity of those mutualistic beneficial microorganisms and accelerating those microbial processes, which augment the availability of nutrients that can then be easily assimilated and absorbed by the plants. Malusá and Vassilev [106] proposed that a biofertilizer is the formulated product containing one or more microorganisms that enhance the nutrient status (the growth and yield) of the plants by either replacing soil nutrients and/or by making nutrients more available to plants and/or by increasing plant access to nutrients.

Biofertilizer products are usually based on the plant growth-promoting microorganisms (PGPM). The PGPM can be classified into three dominant groups of microorganisms: arbuscular mycorrhizal fungi (AMF) [107], plant growth promoting rhizobacteria (PGPR) [108], and nitrogen fixing rhizobia [109], which are deemed to be beneficial to plant growth and nutrition. However, it has been reported that PGPR have been used worldwide as biofertilizers, contributing to increased crop yields and soil fertility. Hence, with the potential contribution of the PGPR, this leads to sustained agriculture and forestry [110].

Previous studies show that a biofertilizer prepared by combining PGPR with composts could enhance growth-promoting effects and bio-control of plants [111]. *Bacillus* spp. [112] and *Pseodomonas* spp. [113] are two PGPR that have been reported to be effective bio-control agents. Among these bacteria species, *Bacillus subtilis*, *Basillus amyloliquefacients*, and *Bacillus cereus* are the most effective species at controlling plant diseases through various mechanisms [114]. The ability to form endospores allows PGPR, especially *Bacillus* spp. and *Pseodomonas* spp., to survive in a wide range of environmental conditions, thus facilitating the effective formulation of biofertilizer [115].

Sufficient densities of PGPR in biofertilizer provide a beneficial role in creating a proper rhizosphere for plant growth and converting nutritionally important elements through biological process, for example increasing the availability of N, P, K, as well as inhibiting pathogen growth [104,116]. The high availability of N, P, and K could enhance soil fertility, improve antagonistic isolates’ bio-control effects, and extend microorganisms’ survival rates in soil [117].

PGPR can be classified as biofertilizers when they act as a plant nourishment and enrichment source that would replenish or reconstruct the nutrient cycle between the soil, plant roots, and microorganisms present. The catch here is whether the “living” biofertilizers used could be self-sustaining or would need to be re-applied to soil on a continual basis, and also whether excessive usage would destabilize the microorganism interaction in the soil.

## 6. Role of Nanotechnology for Agricultural Sustainability

The application of modern technologies such as nanotechnology has tremendous potential to revolutionize the agricultural industry. Nanoagriculture, which currently focuses on target farming that involves the use of nanosized particles such as nanofertilizer, offers exclusive tools for improving the productivity of the crop plants through efficient nutrients uptake by the plants [118]. The unique properties of nanosized particles with respect to their physical, chemical, and biological properties compared to those at a larger scale provide the potential to protect plants, detect plant diseases, monitor plant growth, enhance food quality, increase food production, and reduce waste.

The vast efficiency of nanofertilizers compared to ordinary fertilizers has been proven as they reduce nitrogen loss due to leeching, emissions, and long-term incorporation by soil microorganisms [119]. Furthermore, Suman [120] has proven the advantage of using nanofertilizers by showing that controlled release fertilizers may also improve the soil by decreasing the toxic effects associated with the over-application of traditional chemical fertilizers [120].

PGPR usage as fertilizer by conventional methods is not effective as 90% are lost to the air during application, they are intolerant to the environment (heat, UV radiation, *etc.*), and, as run-off, they affect application costs to the farmer. Nanoencapsulation technology could be used as a versatile tool to protect PGPR, enhancing their service life and dispersion in fertilizer formulation and allowing the controlled release of the PGPR.

## 7. Conclusions

As long as the human population continues to increase, the world will have to withstand the escalating demand for food. Seven decades ago, the Green Revolution increased agricultural production globally, saving about one billion people from starvation and undernourishment; it triggered the development of chemical fertilizers, along with other advances. Since the dawn of civilization, we human beings have been involved in various actions that directly or indirectly impacted on our ecosystem, whether for good or bad. Demand, soon, is catalyzed by greed to increase the crop yield, which has resulted in the overexploitation of the soil ecosystem. This has to be put to rest; the conventional crop approach cannot be practiced anymore since anthropogenic activities such as intensive agriculture, crop monocultures, and the use of agrochemicals are grave concerns and disturb the ecosystem.

Considering the good impact of PGPR in terms of biofertilization, biocontrol, and bioremediation, all of which exert a positive influence on crop productivity and ecosystem functioning, encouragement should be given to its implementation in agriculture. Hoping for the betterment of technology in developing successful research and development, PGPR use will surely become a reality and will be instrumental to crucial processes that ensure the stability and productivity of agro-ecosystems, thus leading us towards an ideal agricultural system.

Nanotechnology inclusion in the agricultural sector should be intensified to reduce the damages to the ecosystem and meet global crop demand. Over the past decades, promising results and applications have already been developed in the area of delivery of fertilizers, pesticides, and genetic material for plant transformation. Based on that, gigantic effort is needed to develop the aspect of nanotechnology with plant growth promoting bacteria. Finding the unique nanomaterials used to incorporate these mutualistic bacteria might prove to be a hard task but it is not impossible. Thus, nanotechnology has all the tools needed to improve the current biofertilizers used to support and uplift agricultural sustainability globally.

## Figures and Tables

**Table 1:** List of plant growth promotion rhizobacteria.

PGPR	PGPR Mechanisms	Crops	Application Mode	Observation/Findings	Ref.
*Azoarcus*	Nitrogen fixation	rice	Plants were grown gnotobiotically with a mutant of strain BH72 expressing the b-glucuronidase gene constitutively.	The presence of *Azoarcus* in the stele, especially in the stelar tissue of culms, suggests that these bacteria might spread systemically *in situ*, and underline their endophytic life style.	[27]
*Azobacter*	Cytokinin synthesis	Cucumber	-	-	[28]
*Azorhizobium*	Nitrogen fixation	Wheat	2 mL of rhizobial culture were added four times to each wheat plant, once during the planting of the seeds, and subsequently three times at one-week intervals.	Five weeks after inoculation with *A. caulinodans* IRBG314, there were approximately five times more short lateral roots, each up to 3 mm in length, present on inoculated wheat.	[29]
*Azospirillum*	Nitrogen fixation	sugar cane	-	-	[30,31,32,33]
*Azotobacter*	Nitrogen fixation	Wheat, barley, oats, rice, sunflowers, maize, line, beetroot, tobacco, tea, coffee and coconuts	-	-	[34]
*Bacillus*	Auxin synthesis	Potato	Seed-dipping (108 mL^−1^ cfu)	Both the strains enhanced the auxin content of inoculated plants up to 71.4% and 433%, respectively, as compared to non-inoculated plants.	[35]
*Bacillus*	Cytokinin synthesis	Cucumber	Seed-dipping 106 cells/mL (106 CFU/mL)	Cucumber seedlings subjected to bacterization had well developed lateral roots.	[36]
*Bacillus*	Gibberelin synthesis	Pepper	-	-	[37]
*Bacillus*	Potassium solubilization	pepper, cucumber	Seedling was inoculated with 1 mL of inoculum containing around 108 cells.	The results showed that there was a relatively higher availability of P and K in soils planted with pepper than with cucumber.	[38,39]
*Bacillus*	Induction of plant stress resistance	Peanuts Maize	Plants were inoculated with 1 mL of a 108 cfu suspension Seed-dipping for 30 min	Increasing salt concentrations, biological N fixation may be competitive, becoming a more economic and sustainable alternative to chemical fertilization. The bacterial inoculants increased the total N, P, and K contents of the shoot and root of maize in calcisol soil from 16% to 85% significantly as compared to the control counterpart.	[40,41]
*Bacillus*	Antibiotic production	Alfalfa	Seedling was inoculated	Filtrates of cultures suppressed alfalfa disease caused by *P. medicaginis* and inhibited the growth of the pathogen in an agar plate assay.	[42]
*Bacillus*	Siderophore production	Maize, pepper	-	-	[43]
*Beijerinckia*	Nitrogen fixation	Sugar cane	-	-	[30,44]
*Burkholderia*	Nitrogen fixation	Rice	-	-	[45,46]
*Chryseobacterium*	Siderophore production	Tomato	Soil drenched	Siderophore production increased as bacterial biomass increased after 16 h of culture	[47]
*Frankia*	Nitrogen fixation	*Alnus*	-	-	[48]
*Gluconacetobacter*	Nitrogen fixation	Sugar cane	Root-dipping of seedlings for 1 h	The endophytic establishment of *G. diazotrophicus* within stems of sugarcane was confirmed by the scanning electron microscopy.	[49]
*Herbaspirillum*	Nitrogen fixation	rice	Seed was inoculated	GFP-tagged cells of *Herbaspirillum* sp. strain B501*gfp*1 were apparently localized in intercellular spaces of shoot tissues of 7-day-old seedlings of *O. officinalis* W0012.	[50]
*Mycobacterium*	Induction of plant stress resistance	Maize	-	-	[40]
*Paenibacillus*	Indole acetic acid synthesis	Lodgepole pine	-	-	[51]
*Paenibacillus*	Potassium solubilization	Black pepper	-	-	[52]
*Phyllobacterium*	Phosphate solubilization	Strawberries	The strawberry seedlings were inoculated with 1 mL of 108 CFU/mL suspensions.	Strain PEPV15 was able to solubilize moderate amounts of phosphate (5mm radius around the colonies).	[53]
*Phyllobacterium*	Siderophore production	Strawberries	The strawberry seedlings were inoculated with 1 mL of 108 CFU/mL suspensions.	The strain grew on the CAS indicator medium where the colonies were surrounded by a yellow-orange halo (3.5 mm radius around colonies) indicative of the siderophore production.	[53]
*Pseudomonas*	Chitinase and β-glucanases production	Several crops	-	-	[54]
*Pseudomonas*	ACC deaminase synthesis	Mung beans, wheat	-	-	[55,56]
*Pseudomonas*	Induction of plant stress resistance	Cotton, Maize	-	-	[40,57]
*Pseudomonas*	Antibiotic production	Wheat	-	-	[58]
*Pseudomonas*	Chitinase and β-glucanases production	Pigeon pea	The method of Weller and Cook (1983) was adopted for seed bacterization	P. fluorescens LPK2 and S. fredii KCC5 showed chitinase activity on chitinase minimal medium. b-1,3-glucanase activity was more pronounced in the fluorescent pseudomonads strains.	[59]
*Pseudomonas*	Siderophore production	Potato, maize	-	-	[43]
*Rhizobia*	Nitrogen fixation	Legumes	-	-	[60]
*Rhizobia*	Induction of plant stress resistance	Peanuts	-	-	[41]
*Rhizobia*	Hydrogen Cyanide Production	Legumes	-	-	[61]
*Rhizobium*	Nitrogen fixation	Rice	-	-	[62]
*Rhizobium*	Indole acetic acid synthesis	Pepper, tomato, lettuce, carrot	Seed Inoculation Seedlings were inoculated with 250 µL plant^−1^ of a bacterial suspension with a turbidity of 5 in McFarland standards (1.5 × 109 CFUmL^−1^).	The dry weight of the inoculated seedlings (shoots and roots) was more than twice with respect to the un-inoculated seedlings. Concentrations of N, P, and Ca were significantly higher in inoculated plants, indicating that they had higher potential for nutrient uptake than control plants.	[63,64]
*Rhizobium*	ACC deaminase synthesis	Pepper, tomato mung beans,	-	-	[55,63]
*Rhizobium*	Siderophore production	Tomato, pepper, Carrot, lettuce,	Seed Inoculation Seedlings were inoculated with 250 lL plant^−1^ of a bacterial suspension with a turbidity of 5 in McFarland standards (1.5 × 109 CFU/mL^−1^).	The colonies of strain TPV08 were surrounded by a yellow-orange halo (3.5 mm radium around colonies) indicative of siderophore production.	[63,64]
*Sinorhizobium*	Chitinase and β-glucanases production	Pigeon pea	-	-	[59]
*Sphingomonas*	Gibberelin synthesis	Tomato	-	-	[65]
*Streptomyces*	Indole acetic acid synthesis	Indian lilac	-	-	[66]
*Streptomyces*	Siderophore production	Indian lilac	-	-	[66]
